# Stereotactic Radiosurgery for Primary Central Nervous System Lymphoma

**DOI:** 10.7759/cureus.34817

**Published:** 2023-02-09

**Authors:** Susan Y Wu, Steve E Braunstein, James L Rubenstein, Penny K Sneed

**Affiliations:** 1 Department of Radiation Oncology, The University of Texas MD Anderson Cancer Center, Houston, USA; 2 Department of Radiation Oncology, University of California San Francisco, San Francisco, USA; 3 Department of Medicine, University of California San Francisco, San Francisco, USA

**Keywords:** stereotactic radiosurgery (cyberknife®), gamma knife (gk) radiosurgery, cns lymphoma, stereotactic radiosurgery, primary central nervous system lymphoma (pcnsl)

## Abstract

Background

Primary central nervous system lymphoma (PCNSL) is rare, with a treatment backbone that typically includes high-dose methotrexate-based chemotherapy, with radiation often reserved for persistent or progressive disease. In this study, we report the outcomes of stereotactic radiosurgery (SRS) in patients with PCNSL to potentially defer whole brain radiotherapy (WBRT) or as salvage after WBRT.

Methodology

We performed a single-institution, retrospective review of 20 patients with PCNSL who received single-fraction or fractionated SRS to 32 lesions between September 1992 and July 2019.

Results

The median age at SRS was 67 years (interquartile range (IQR) = 56-74 years). The median Karnofsky Performance Status (KPS) at SRS was 80 (IQR = 50-80). In total, 18 (90%) patients received methotrexate-based chemotherapy prior to SRS, with a median of eight cycles (IQR = 5-10). A total of 10 patients received SRS for recurrent disease after chemotherapy and/or WBRT, nine patients received SRS for the persistent disease after chemotherapy alone, and one patient received up-front SRS. Overall, five patients received SRS following WBRT. The median SRS dose was 16 Gy (IQR = 14-22.5 Gy) in one fraction (IQR = 1-5 fractions). Eight patients (40%) were treated with consolidative pomalidomide or lenalidomide following SRS. The local control rate was 100% (32/32 lesions at a median follow-up of 15 months). In total, 13 of 16 (81%) patients with available follow-up experienced distant brain recurrence. The median time to distant failure following SRS was 10 months (IQR = 1-16 months). Three patients received salvage SRS, and three patients received salvage WBRT. The median overall survival from diagnosis was 39 months (95% confidence interval = 24-54 months). KPS at the time of SRS was significantly correlated with time to progression (p = 0.002). The use of lenalidomide or pomalidomide after SRS was associated with improved overall survival after SRS (three vs. 14 months, p = 0.035). Consolidative etoposide and cytarabine after initial methotrexate-based chemotherapy was also associated with improved survival following SRS (eight vs. 47 months, p = 0.028).

Conclusions

SRS offers effective local tumor control for patients with PCNSL; however, the majority of patients experience distant progression. SRS may have a role in the salvage setting for patients with recurrence after WBRT, or allow deferral of WBRT in select patients, although systemic therapy appears to strongly influence outcomes in this cohort.

## Introduction

Primary central nervous system lymphoma (PCNSL) is rare, constituting approximately 2% of all primary central nervous system (CNS) malignancies in the United States. The majority of cases occur in immunocompetent patients, with a median age of 66 years and a slight male predominance (1.04:1) [1,2]. Histologically, these tumors are typically diffuse large B-cell lymphomas and are negative for Epstein-Barr virus (EBV). In patients with human immunodeficiency virus (HIV)/acquired immunodeficiency syndrome (AIDS), PCNSL typically presents earlier and is associated with EBV. With the advent of highly active antiretroviral therapy, the incidence of AIDS-associated PCNSL has declined.

Historically, treatment for PCNSL has been derived primarily from phase II trials and retrospective series, with a backbone of high-dose methotrexate (HD-MTX)-based chemotherapy, with or without whole brain radiotherapy (WBRT) [3]. To date, the only randomized phase III trial in PCNSL addressed the role of consolidative WBRT following HD-MTX-based chemotherapy and demonstrated improvement in progression-free survival (PFS) but not overall survival (OS) with WBRT [4]. Given significant neurotoxicity in patients receiving HD-MTX and WBRT [5], which may be fatal in up to 10% of patients [6], treatment with chemotherapy alone and deferring WBRT is an appealing strategy, particularly in those with a complete response (CR) to chemotherapy [3,5,7].

However, even following a CR to HD-MTX-based chemotherapy, about one-third of patients relapse, and the prognosis for these patients is poor [8,9]. Several retrospective series have suggested that the use of stereotactic radiosurgery (SRS) immediately following HD-MTX or in the salvage setting for persistent or progressive disease following chemotherapy is well tolerated while offering high rates of local control [10,11]. This study describes our institutional experience with SRS in patients with PCNSL.

## Materials and methods

Patient information

We performed a retrospective, single-institution review of 20 patients with histologically confirmed PCNSL who received single-fraction or fractionated SRS from September 1992 to July 2019. A total of 32 lesions were treated. Patient characteristics such as age, gender, prior treatment, and Karnofsky Performance Status (KPS) were abstracted from medical records and vital status was confirmed with our institutional tumor registry. All patients provided informed consent for treatment. Consent for this retrospective review was waived by the institutional review board (IRB #18-26489).

All patients considered for SRS were reviewed by a multidisciplinary tumor board. Recommendation for single-fraction Gamma Knife SRS or fractionated SRS was based on a combination of factors, including prior treatment, patient performance status, lesion size, and location, with larger lesions in more eloquent areas considered for a more fractionated approach. The prescription dose was at the discretion of the treating physician but similarly took into account target size and location.

Gamma Knife stereotactic radiosurgery procedure

Patients who received single-fraction SRS were treated using a Leksell Gamma Knife (Elekta, Stockholm, Sweden) with a stereotactic frame for immobilization. Following frame placement by a neurosurgeon with local anesthetic, patients underwent high-resolution, thin-slice MRI with contrast to allow better target delineation in the treatment planning software (GammaPlan). No expansion was added to the gross tumor volume (GTV). Treatment plans were approved by both a radiation oncologist and neurosurgeon prior to delivery. The prescription dose and treatment volume were recorded for each treated lesion.

Linac-based stereotactic radiosurgery procedure

**Figure 1:**
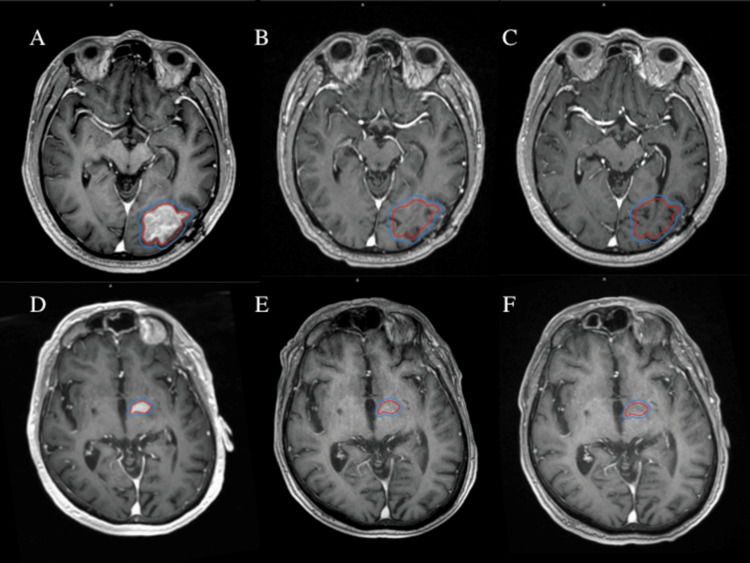
Representative axial T1 post-contrast MRI images demonstrating target delineation for linac-based radiosurgery in the treatment of PCNSL and response following SRS. GTV is contoured in red. PTV is contoured in blue. Patient one is a 46-year-old man with relapsed PCNSL following complete response to HD-MTX: (A) Planning MRI with a 3 mm expansion from GTV to PTV, prescription dose 25 Gy in five fractions. (B) Follow-up MRI images at two months and (C) four months following treatment. Patient two is a 74-year-old man with progressive PCNSL following HD-MTX, rituximab, and pomalidomide, as well as SRS to two lesions one year prior: (D) Planning MRI with 2 mm GTV to PTV expansion, prescription dose 25 Gy in five fractions, follow-up MRI images at (E) two months and (F) five months. Both patients had resolution of enhancing disease by their second follow-up MRI (C & F). PCNSL = primary central nervous system lymphoma; SRS = stereotactic radiosurgery; GTV = gross tumor volume; PTV = planning target volume; HD-MTX = high-dose methotrexate

Patients who received fractionated SRS were treated either on a TrueBeam linear accelerator (Varian Medical Systems, Inc., Palo Alto, CA) with a stereotactic thermoplastic mask for immobilization and cone-beam CT with ExacTrac for image guidance, or using Cyberknife (Accuray, Sunnyvale, CA) with 6D skull-tracking. Diagnostic MRI images were fused to planning CT images and lesions were contoured in RayStation (RaySearch Laboratories, Stockholm, Sweden) or MIM (version 6.8.3, MIM Software Inc., Cleveland, OH). The GTV to planning target volume (PTV) expansion was generally 2 mm, but varied depending on the size and location of the lesion (Figure 1).

Follow-up

Patients underwent follow-up MRI initially one to two months following treatment, and then every two to three months. A subset of patients received consolidative lenalidomide or pomalidomide, in which case the schedule of follow-up imaging was at the discretion of their treating medical oncologist.

Statistical analysis

Chi-square and Mann-Whitney U tests were used in the univariate analysis of predictors of outcomes. The Kaplan-Meier method was used to describe freedom from distant failure (FFDF) and OS and was calculated from the time of SRS to death or last follow-up. Due to a limited number of events, Mann-Whitney U tests were also used to compare median survival between patients receiving different adjuvant therapies. A two-sided p-value ≤0.05 was considered significant. Statistics were performed using SPSS version 25 (IBM Corp., Armonk, NY, USA).

## Results

A total of 20 immunocompetent patients were treated between September 1992 and July 2019 (Table 1). In total, 11 (55%) patients were female. The median age at diagnosis was 65 years (interquartile range (IQR) = 55-73 years), and the median age at SRS was 67 years (IQR = 56-74 years). The median KPS at SRS was 80 (IQR = 50-80). All patients underwent histologic confirmation of PCNSL. A total of 18 (90%) patients received methotrexate (MTX)-based chemotherapy prior to SRS, with a median of eight cycles (IQR = 5-10) (Table 2). The most common systemic therapy regimen was HD-MTX, rituximab, and temozolomide in 10 (50%) patients. Of the two patients who did not receive up-front chemotherapy, one had poor renal function, and the other received up-front WBRT at an outside institution. Three (15%) patients received consolidative etoposide and cytarabine (EA) following initial MTX-based chemotherapy.

**Table 1:** Patient characteristics *: This 77-year-old patient is 11 years from diagnosis with a KPS of 70 and has not received WBRT. IQR = interquartile range; SRS = stereotactic radiosurgery; KPS = Karnofsky Performance Status; WBRT = whole brain radiotherapy

Characteristic	Median (IQR) or n (% of 20)
Age at diagnosis	65 (55–73)
Age at SRS	67 (56–74).
Female	11 (55%)
Ethnicity	
White	14 (70%)
Asian	6 (30%)
KPS at SRS	80 (50–80)
Multiple lesions treated	4 (20%)
Multiple courses of SRS	
2 courses	2 (10%)
4 courses*	1 (5%)
Indication for SRS	
Up-front	1 (5%)
Recurrent disease	10 (50%)
Persistent or progressive	9 (45%)

**Table 2:** Treatment characteristics. *: Lesion-based analysis (n = 32). HD-MTX = high-dose methotrexate; IQR = interquartile range; MTX = methotrexate; EA = etoposide and cytarabine; R = rituximab; TMZ = temozolomide; WBRT = whole brain radiotherapy; SRS = stereotactic radiosurgery; PTV = planning target volume

Treatment prior to SRS	Median (IQR) or N (% of 20 unless otherwise specified)
HD-MTX-based chemotherapy	18 (90%)
MTX + R	2 (10%)
MTX + R + TMZ	10 (50%)
Median cycles of HD-MTX	8 (5–10)
Consolidative EA	3 (15%)
WBRT	5 (25%)
SRS characteristics*	
Single-fraction SRS	21 (66%)
PTV (cc)	1.9 (0.6-5.8)
Prescription dose (Gy)	14.5 Gy (14-16)
Three-fraction SRS	3 (9%)
PTV (cc)	2.8 (0.9-2.8cc)
Prescription dose (Gy)	22.5 Gy (22.5-22.5)
Five-fraction SRS	
PTV (cc)	31.1 (16.5-45.1)
Prescription dose (Gy)	25 (23.5–25.0)
Post-SRS treatment	
Lenalidomide or pomalidomide	8 (40%)
Additional (salvage) SRS	3 (15%)
WBRT	3 (15%)

In total, 10 (50%) patients received SRS in the setting of recurrent disease (six with prior CR to HD-MTX-based chemotherapy), while nine (45%) patients underwent SRS for persistent disease following chemotherapy. One patient received up-front SRS as he was not a candidate for chemotherapy and wanted to avoid WBRT. Five (25%) patients received SRS for progression after WBRT. The median interval from WBRT to salvage SRS was 10 months (IQR = 5-18 months).

A total of 32 lesions were treated. Overall, 21 (66%) lesions were treated with single-fraction SRS, with a median prescription dose of 14.5 Gy in one fraction (IQR = 14-16 Gy). Eleven (34%) lesions were treated with fractionated SRS, with a median prescription dose of 25 Gy (IQR = 22.5-25 Gy) in five fractions (IQR = 3-5 fractions). In total, 23 (72%) lesions were treated during a patient’s first course of SRS. Of the remaining nine lesions, three (9%) lesions were part of a second course, two (6%) lesions were treated during a third course, and four (13%) lesions were treated as part of a fourth course. The median PTV for lesions treated in a single fraction was 1.9 cc (IQR = 0.6-5.8 cc), while the median PTV for lesions treated with fractionated SRS was larger at 17 cc (IQR = 4.8-39 cc) (p = 0.007) (Table 2).

**Figure 2:**
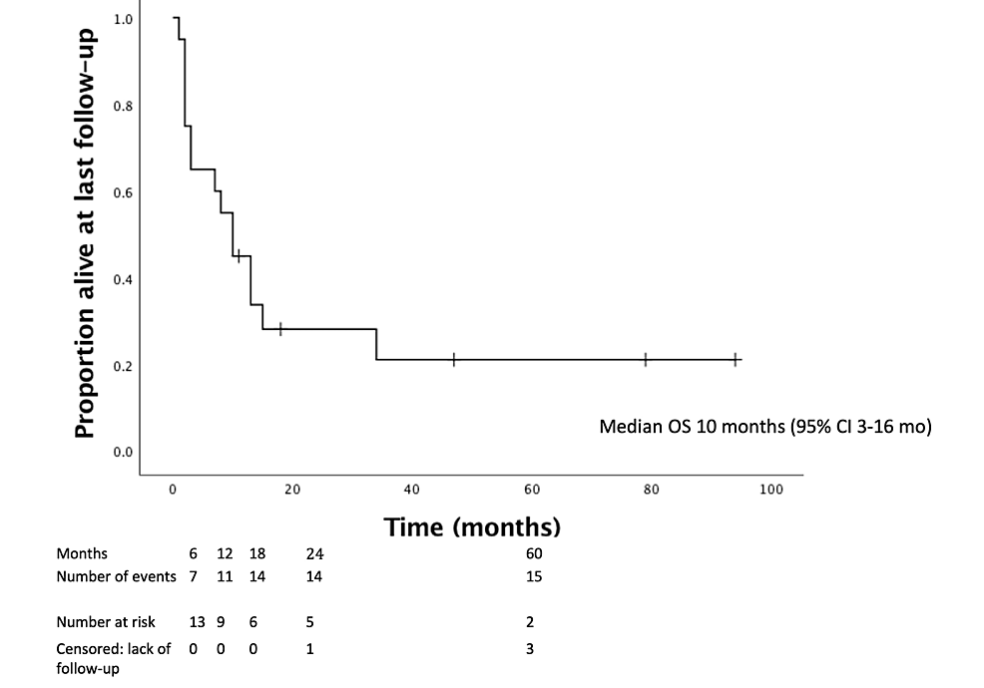
OS following SRS. OS = overall survival; SRS = stereotactic radiosurgery; CI = confidence interval

The median OS from the time of diagnosis was 39 months (95% confidence interval (CI) = 24-54 months). The median time from diagnosis to SRS was 16 months (IQR = 5-43 months). The median follow-up was 58 months (IQR = 29-100 months) among the five living patients (25%) and 15 months overall (IQR = 9-46 months). The median OS following SRS was 10 months (95% CI = 3-16 months) (Figure 2). Three- and six-month OS after SRS was 75% (15/20) and 65% (13/20), respectively.

Local control per lesion was 100% (32/32). There were no instances of radiographic or clinically apparent radiation necrosis following SRS. Two patients did have substantial T2/fluid-attenuated inversion recovery signal abnormalities following SRS, however, without enhancement or restricted diffusion and without associated symptoms.

**Figure 3:**
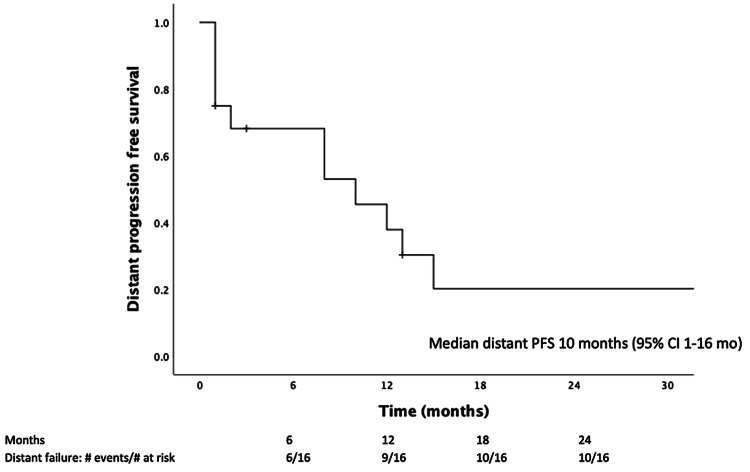
Distant PFS following SRS. PFS = progression-free survival; SRS = stereotactic radiosurgery; CI = confidence interval

Of the 16 patients with available follow-up, 13 (81%) experienced distant brain recurrence (Figure 3). Five (31%) patients experienced distant recurrence within three months of SRS. Three (15%) patients underwent additional salvage SRS for distant brain recurrence; two patients received a second course of SRS four months following their first course, and one patient received three additional courses of SRS at three, nine, and 20 months following his first SRS treatment. Three (15%) patients received WBRT for distant failure at one, three, and 28 months following SRS.

**Figure 4:**
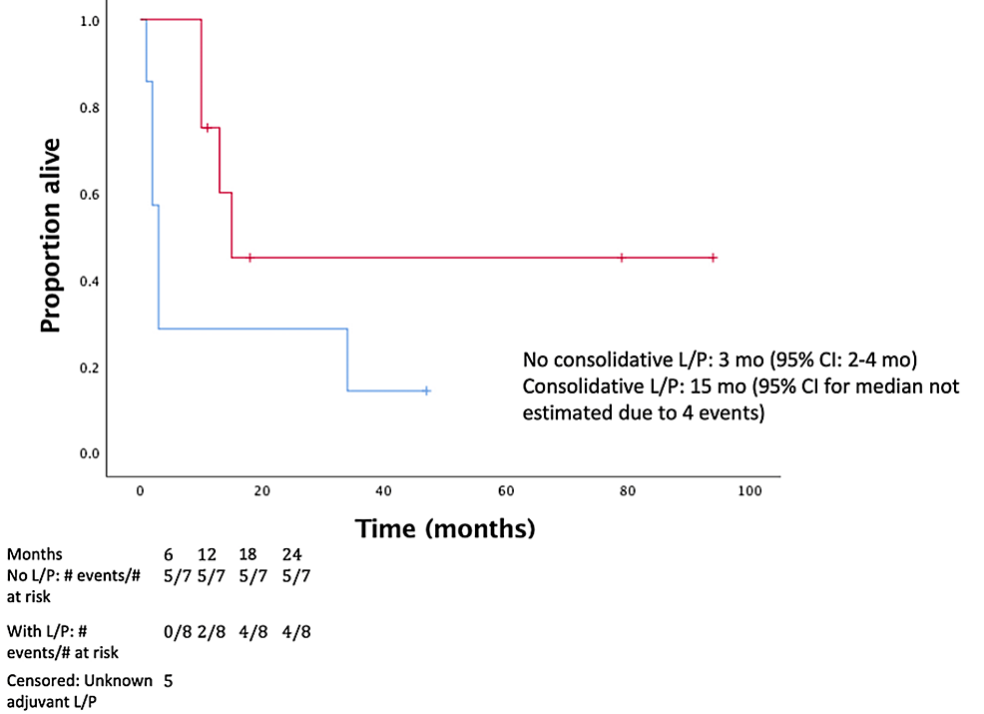
OS in those who received post-SRS L/P (red) and those who did not (blue) (p = 0.035). OS = overall survival; SRS = stereotactic radiosurgery; L/P = lenalidomide/pomalidomide; CI = confidence interval

**Figure 5:**
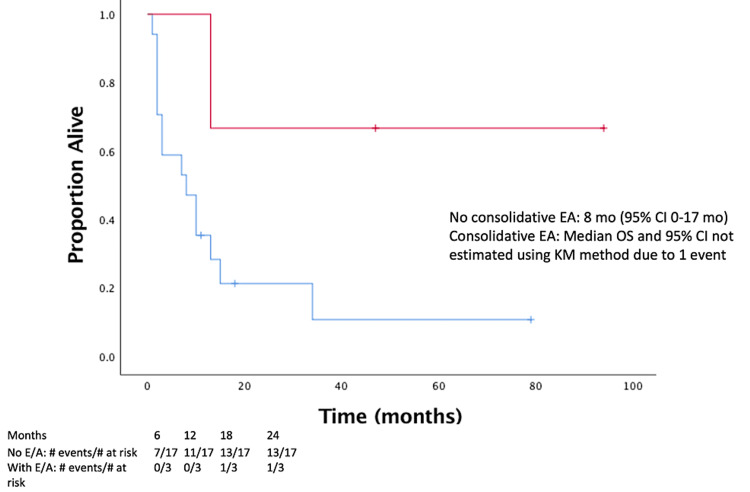
OS in those who received consolidative EA after initial methotrexate-based chemotherapy (red) and those who did not (blue) (p = 0.028). OS = overall survival; EA = etoposide and cytarabine; CI = confidence interval; KM = Kaplan-Meier

KPS at the time of SRS was significantly correlated with time to progression (p = 0.002). Eight (40%) patients were treated with consolidative pomalidomide or lenalidomide following SRS. The use of pomalidomide or lenalidomide after SRS was associated with improved six-month OS after SRS (77% vs. 0%, p = 0.026) and median OS from SRS (three vs. 15 months, p = 0.035) (Figure 4). Consolidative EA, after initial HD-MTX chemotherapy, was associated with improved median OS from SRS (eight vs. 47 months, p = 0.028) (Figure 5). KPS was not associated with receiving pomalidomide or lenalidomide (p = 0.44), or consolidative EA (p = 0.30).

Other clinical variables including gender, age at diagnosis or SRS, cycles of HD-MTX, time since diagnosis, size of lesions treated, and RT dose were not associated with FFDF or OS.

## Discussion

Our study represents a large cohort of patients with PCNSL treated with SRS and demonstrates excellent local control. Despite high rates of distant failure after SRS, there were some long-term survivors. In our cohort, one patient was alive and without evidence of disease at 116 months of follow-up. This suggests that aggressive management with SRS may be a reasonable approach in some patients. Our data also suggest that systemic therapy, particularly consolidative EA or pomalidomide/lenalidomide, may be beneficial and merits further exploration in combination with up-front HD-MTX-based regimens, with or without WBRT.

Due to the concern for microscopic extension and multifocal disease, WBRT has conventionally been the radiation treatment of choice for patients with PCNSL, despite a poor median OS of about 18 months when used alone [12]. The addition of HD-MTX-based chemotherapy prior to WBRT has been shown through several phase II trials to improve median survival, though the optimal chemotherapy regimen continues to be refined. HD-MTX-based systemic therapy regimens, particularly with rituximab and temozolomide, may lead to improved outcomes [3,9].

The only phase III randomized trial in PCNSL demonstrated no OS benefit to consolidative WBRT after chemotherapy, and no PFS or OS benefit in those with a CR to HD-MTX [4]. This finding is also supported by retrospective data [3]. As such, WBRT may reasonably be reserved for salvage therapy, and in that setting, a lower dose of ≤36 Gy may be associated with a lower incidence of neurotoxicity [7]. CALGB 50202 evaluated a combination of HD-MTX, rituximab, and temozolomide for induction chemotherapy, with consolidated EA in those with CR, and demonstrated superior CR, two-year PFS, and two-year OS (66%, 57%, and 70%, respectively) compared to HD-MTX-based polychemotherapy, WBRT, and consolidation cytarabine per RTOG 9310 (58%, 50%, and 64%, respectively) [9,13]. RTOG 0227 demonstrated even higher two-year PFS and OS rates (64% and 81%, respectively) using a combination of HD-MTX with the addition of rituximab and temozolomide, followed by hyperfractionated WBRT and consolidative temozolomide [14]. In patients aged 60 years or younger, phase II data comparing WBRT (40 Gy in 20 fractions) with autologous stem cell transplantation (ASCT) after induction chemotherapy demonstrated no statistically significant difference in four-year OS (64% in the WBRT arm, 66% in the ASCT arm), though PFS and cognitive function endpoints favored the ASCT arm [15]. Data regarding dose-reduced WBRT in patients with a CR to chemotherapy is mixed, with a two-year OS of 77%, as described in Morris et al. compared to 49% described in Adhikari et al. [16,17]. It is unclear if the improved survival in Morris et al. [17] was due to consolidative cytarabine after radiation or uniform use of cetuximab. Across these studies, the majority of patients eventually experience progression despite WBRT.

SRS has been used in patients with PCNSL with the goal of avoiding, or perhaps deferring, whole brain radiation given significant neurotoxicity, particularly among older patients [6]. A prospective, single-institution, observational study suggests improved OS with the addition of SRS to HD-MTX in newly diagnosed patients (median OS = 47.6 vs. 26.8 months, p = 0.0034) [18]. Local control, albeit at three to eight weeks of follow-up, was 100% in this study; however, the rate of distant failure was not included. Hirono et al. published results of consolidative SRS following HD-MTX in the definitive setting with a high rate of local control at 86%. The two-year OS rate in this study of 75% is similar to results seen with chemotherapy with or without WBRT [9,10].

Similar trends have been observed following SRS for relapsed or refractory disease, with short intervals to distant progression despite high rates of local control. Shin et al. reported a series of 23 patients treated with salvage SRS, most with prior HD-MTX-based chemotherapy and almost half with prior WBRT, and demonstrated CR in over half of patients with six-month local control of 91% and PFS of 81% [11]. Kenai et al. reported a series of 22 patients, 18 of whom had received prior treatment, and demonstrated 100% local control at 19 months follow-up, with distant failure in 45% of patients and a median OS of 38 months [19]. Similarly, in our series, local control was high (100%, 32/32 lesions), but a greater proportion of patients (81%) experienced distant failure. Although the cohort described in Kenai et al. [19] was also heavily pretreated, it is possible that the patients in our cohort, 50% of whom progressed or recurred following HD-MTX, rituximab, and TMZ, may have had more unfavorable biology. In our series, 31% of patients with available follow-up experienced distant progression within three months of SRS, which was not associated with the use of lenalidomide/pomalidomide (p = 0.88) or consolidative EA (p = 0.29) following SRS. Furthermore, Hirono et al. and Alvarez-Pinzon et al. reported longer OS (48 and 52 months, respectively) with HD-MTX-based systemic therapy and SRS as first-line treatment, compared to about 40 months in our cohort, suggesting that reserving SRS for salvage may not be the optimal sequence of treatment [10,18]. It must also be noted, however, that the estimated median OS reported in RTOG 0227 was significantly longer than both these series of up-front chemotherapy and SRS at 7.5 years [14]. This suggests that WBRT should remain the standard of care, particularly for patients with an incomplete response to chemotherapy [4].

In patients heavily pretreated with HD-MTX and radiation, there are few remaining salvage options such as re-challenge with HD-MTX [3], alternative chemotherapy [20,21], or ASCT [22]. Lenalidomide, a thalidomide derivative, has been shown to induce a CR in some heavily pretreated patients and is well tolerated, making it an attractive option in patients with relapsed or refractory PCNSL [23]. The use of lenalidomide or pomalidomide after salvage SRS is a common practice at our institution, and based on the data presented, may be associated with improved outcomes after SRS, though numbers are quite small.

Our data are limited in that they represent a heterogeneous, single-institution, retrospective series spanning over 27 years. Due to the time frame in which patients were treated, we also lacked detailed follow-up information for some patients and are unable to comment robustly on clinical improvement or deterioration following SRS. While data from the brain metastasis literature suggests decreased rates of cognitive decline in patients treated with SRS alone compared to WBRT alone [24] or SRS and WBRT [25], we must note that our data do not include the assessment of neurocognitive function in this retrospective cohort. Ultimately, data regarding the use of SRS in PCNSL remain limited. To date, only four series have been published including 20 or more patients, two of which were in the up-front setting [10,11,18,19]. Given the relative rarity of PCNSL, these findings may warrant further prospective investigation at the cooperative group level.

## Conclusions

While SRS offers effective local tumor control in patients with PCNSL, prognosis remains poor due to a high rate of distant progression after treatment. Further research is necessary to evaluate the role of radiation following treatment with HD-MTX and rituximab-based systemic therapy, as well as the efficacy of salvage or consolidative lenalidomide or pomalidomide. WBRT should still be considered the standard of care in young patients with persistent or progressive disease after chemotherapy.
